# The ethical framework for performing research with rare inherited neurometabolic disease patients

**DOI:** 10.1007/s00431-017-2852-9

**Published:** 2017-01-16

**Authors:** Viviana Giannuzzi, Hugo Devlieger, Lucia Margari, Viveca Lena Odlind, Lamis Ragab, Cinzia Maria Bellettato, Francesca D’Avanzo, Christina Lampe, Linda Cassis, Elisenda Cortès-Saladelafont, Ángels Garcia Cazorla, Ivo Barić, Ljerka Cvitanović-Šojat, Ksenija Fumić, Christine I Dali, Franco Bartoloni, Fedele Bonifazi, Maurizio Scarpa, Adriana Ceci

**Affiliations:** 1 Fondazione Per la Ricerca Farmacologica Gianni Benzi Onlus, Via Abate Eustasio 30, 70010 Valenzano, BA Italy; 2 Department of Development and Regeneration, University of Leuven, O&N IV Herestraat 49-box 805, 3000 Leuven, Belgium; 3 Department of Basic Medical Sciences, Neuroscience and Sense Organs, “Aldo Moro” University of Bari, Piazza Giulio Cesare 11, 70124 Bari, Italy; 4 Department of Obstetrics and Gynaecology, Uppsala Universitet, 751 85 Uppsala, SE Sweden; 5 Cairo University, Al Orman Guiza, Giza, 12613 Egypt; 6 Brains for Brain Foundation – onlus, Padova, Italy; 7 Department of Pediatric and Adolescent Medicine, Centre for Rare Diseases, Horst Schmidt Klinik Wiesbaden, Wiesbaden, Germany; 8 Neurology, Gastroenterology Pathology and Clinical Biochemistry Departments, IRP-HSJD and CIBERER, Barcelona, Spain; 9 University Hospital Centre Zagreb, Zagreb, Croatia; 10 School of Medicine, University of Zagreb, Zagreb, Croatia; 11 Department of Clinical Genetics, Copenhagen University Hospital, Rigshospitalet, Copenhagen, Denmark

**Keywords:** Paediatric, Rare, Genetic disease, Clinical research, Ethics

## Abstract

The need for performing clinical trials to develop well-studied and appropriate medicines for inherited neurometabolic disease patients faces ethical concerns mainly raising from four aspects: the diseases are rare; include young and very young patients; the neurological impairment may compromise the capability to provide ‘consent’; and the genetic nature of the disease leads to further ethical implications. This work is intended to identify the ethical provisions applicable to clinical research involving these patients and to evaluate if these cover the ethical issues. Three searches have been performed on the European regulatory/legal framework, the literature and European Union-funded projects. The European legal framework offers a number of ethical provisions ruling the clinical research on paediatric, rare, inherited diseases with neurological symptoms. In the literature, relevant publications deal with informed consent, newborn genetic screenings, gene therapy and rights/interests of research participants. Additional information raised from European projects on sharing patients’ data from different countries, the need to fill the gap of the regulatory framework and to improve information to stakeholders and patients/families.

*Conclusion*: Several recommendations and guidelines on ethical aspects are applicable to the inherited neurometabolic disease research in Europe, even though they suffer from the lack of a common ethical approach.

**Table:** 

**What is Known:**
• *When planning and conducting clinical trials, sponsors and researchers know that clinical trials are to be performed according to well-established ethical rules, and patients should be aware about their rights.*
• *In the cases of paediatric patients, vulnerable patients unable to provide consent, genetic diseases’ further rules apply.*
**What is New:**
• *This work discusses which ethical rules apply to ensure protection of patient’s rights if all the above-mentioned features coexist.*
• *This work shows available data and information on how these rules have been applied.*

## Introduction

Inherited NeuroMetabolic Diseases (iNMDs) represent a particularly important group of rare diseases being constituted by genetic metabolic disorders that may impact on the brain from birth (sometimes already in utero) and during the whole developmental period causing mental retardation and/or different neurological signs and symptoms, may be progressive and may ultimately end with irreversible consequences and even in early death [2].

Today, as a result of medical research, major progress has been made. The scenario of the ‘personalised medicine’, based on the premise ‘the right treatment for the right patient at the right time’, is proposing the use of the latest advancements in biological knowledge and technology, such as the completion of the human genome mapping, to improve understanding of inter-individual variability, in order to include them in the medicine developmental process. The research based on ‘omics’ technologies, including pharmacogenetics/pharmacogenomics (PGt/PGx), is producing a very high amount of data. In fact, these approaches have been demonstrated to be useful both in pharmacological treatment and in diagnosis.

However, the number of curative interventions remains low and this low number of approved drugs is strictly connected to the difficulties characterising the research in this field that can be summarised as the following.

First of all, when dealing with *children*, clinical research raises many scientific and ethical concerns, and special provisions should apply [6]. Due to developmental and cognitive limitations and legal incapacity, a child is legally unable to provide informed consent to participate in research that should be done by a legal representative [28]. On the other hand, children are entitled to receive appropriate information about their health and their participation in clinical studies that imply that their views can be sought and taken into account. Challenges and obstacles in accessing the views of children and young people have been identified by the Paediatric Committee (PDCO) and include access, language/ability to express oneself, ethnic and cultural differences, chronological age and maturity, ill-informed or preconceived notions about a child’s level of understanding and maturity [26]. The call for more medical research involving children, as enlightened by the European Directive 2001/20/EC [30] and the Paediatric Regulation [32, 33], has also raised the question whether the law strikes an appropriate balance between the need for clinical trials and the interests and rights of the child.

Secondly, when paediatric patients are affected by rare diseases, difficulties double [5] as children should be considered ‘twice orphan’, and randomised controlled trials, considered as the standard in research design, are even more unfeasible due to the smaller number of patients. It becomes ethically problematic to propose a control arm (in which the investigational product/approach is not provided to a segment of the population) for a study aimed to establish the efficacy of a new product/approach.

The difficulties to propose a control arm are also true for prenatal and neonatal screenings, which represent a fundamental step in the diagnosis of genetic diseases. Screening programs for genetic diseases have multiplied in the last 50 years [50]. Research in this field is often observational with either historical control data or control through comparisons with similar populations without screening [3].

More in general, clinical research on *genetic diseases* raises additional scientific and ethical concerns, including specific ethical procedures for genetic research, collection, storage and access to genetic materials, aims of the use of genetic information, time of archiving genetic material in biobanks, informed consent, special issues dealing with confidentiality and paediatrics.

Genetic testing guides the prevention, clinical management and drug treatment based on genetic susceptibilities [4]. Carriers/patients must consider disclosure of information to others and weigh the right to privacy against common interests [17, 39]. In addition, knowledge of one’s carrier status for recessive genetic diseases is useful primarily in making reproductive decisions. Such decisions are within the private domain of the young adults who are dating, mating and forming new families. The privacy of these decisions may be compromised when parents know the carrier status of their children.

Another aspect to be considered is the use of advanced therapy medicinal products (ATMPs), including gene therapy, and the so-called personalised medicine. Today, for this type of diseases, this represents the most innovative research. For example, when a product is autologous (meaning that it ‘originates from the same patient’), who is the owner of the product? The company or the patient?

Finally, the *neurological impairment* characterising patients affected by iNMDs can lead to mental disability and make both paediatric and adult patients vulnerable (persons relatively or absolutely incapable of protecting their own interests and therefore not capable of giving adequately consent) unable to give their consent or assent. The current legislation [28] defines these patients ‘incapacitated subjects’.

In order to perform well-conducted research in this field, many regulatory/ethical and legal provisions need to be followed by researchers, not limited to good clinical practice [25], to considering all these aspects. For this reason, this work aims the following:

1. To identify the relevant provisions to perform well-conducted research from the ethical point of view in the field of iNMDs, taking into account the issues listed above
2. To evaluate if the existing provisions cover the ethical issues related to:
  - Rare diseases: clinical studies require ad hoc methodological approaches which should minimise the number of patients and ensure the adequateness of results and the statistical power at the same time
  - Children and patients with neurological symptoms: vulnerable patients requiring ad hoc protection
  - Genetic/inherited diseases: specific ethical procedures for genetic research, collection, storage and access to genetic materials, aims of the use of genetic information, time of archiving genetic material in biobanks and informed consent

All these conditions share the important ethical issue concerning the need for studying and making available drugs notwithstanding the scarce economic return coming from their development.

## Methods

In order to reach the aim of this work, the following actions were undertaken:

1. To search for the current ethical provisions applicable in Europe to the iNMD research

The actual European regulatory/ethical and legal framework was reviewed. Other relevant international guidelines and texts were taken into account, as well. The following sources were consulted: EudraLex Volume 10—Clinical trials guidelines; International Conference on Harmonisation (ICH) guidelines; World Health Organization (WHO)—Council for International Organizations of Medical Sciences (CIOMS) guidelines; EC Public Health publications; Council of Europe treaties; World Medical Association publications; UNESCO Declarations; and European Medicines Agency (EMA).

Then, the applicability of retrieved documents in the iNMD field was analysed by considering the specific features of iNMDs, i.e.:

- The rarity of conditions
- The presence of paediatric patients
- The neurological impairment that often compromises their capability to provide the consent or assent
- The genetic nature of the disease

2. To review relevant data from the literature

**Fig. 1:**
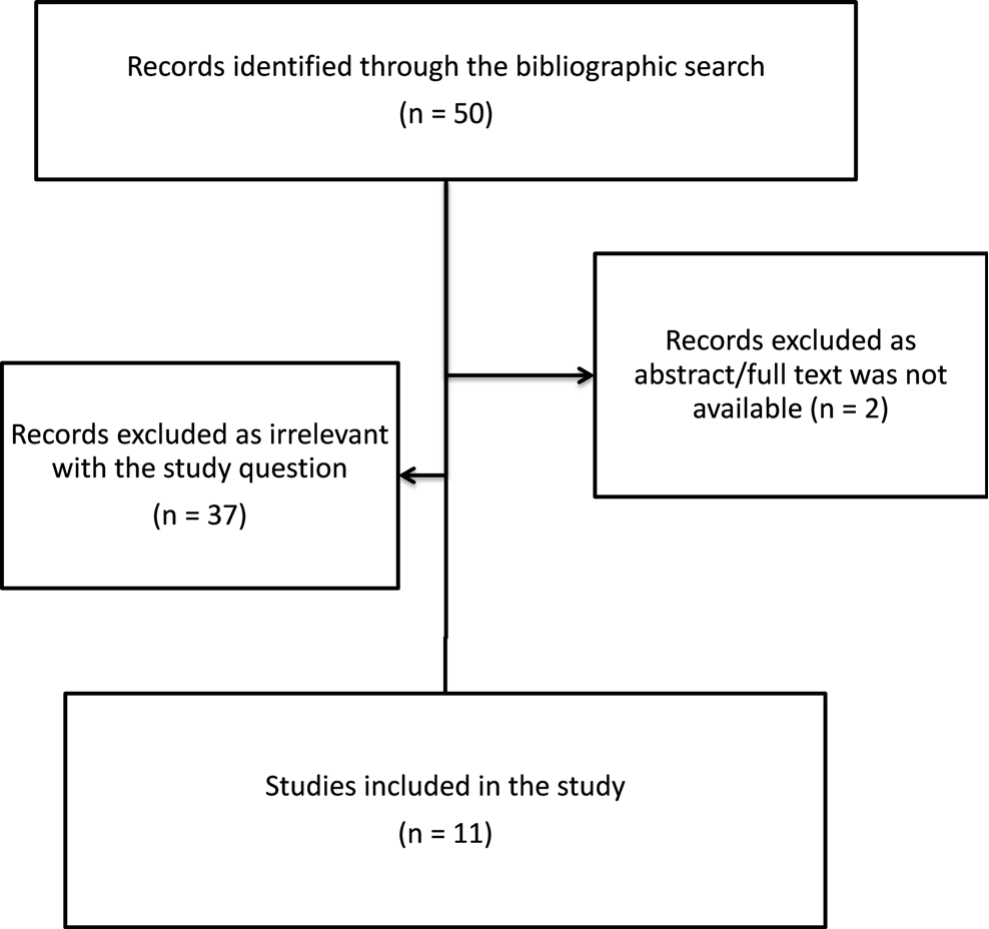
Bibliographic search flow chart

An ad hoc bibliographic search was performed in PubMed (Fig. 1). The publications discussing ethical issues related to research involving iNMD patients and the application of the existing provisions in the field were considered compliant with the search.

The following search strategy was used: (“Brain Diseases, Metabolic, Inborn”[Mesh]) AND “Ethics, Clinical”[Mesh] OR (“Brain Diseases, Metabolic, Inborn”[Mesh]) AND “Ethics, Research”[Mesh]. Only publications in English were considered.

Even in this second step, the rarity of conditions, the inheritance and familiar implications, the presence of paediatric patients and the manifestation of neurologic symptoms which can make paediatric and adult patients unable to provide the consent were focused on.

3. To find relevant results and achievements from European projects

We also looked for data relevant to the aims of this work from already existing EU-funded projects on paediatric and rare disease research. The Community Research and Development Information Service (CORDIS) and Consumers, Health, Agriculture and Food Executive Agency (CHAFEA) were searched by using the following keywords: (paediatric OR rare or genetic) AND disease AND clinical AND research AND ethics. Public websites were consulted, where existing.

## Results

### The current ethical provisions applicable in Europe to inherited neurometabolic diseases

The actual European regulatory/ethical and legal framework offers document ruling, in general, the clinical research including ethical provisions applicable to the iNMD research: Regulation (EU) 536/2014 [28]; Directive 2005/28/EC [20]; ICH Guideline for Good Clinical Practice [25]; EMA Reflection paper on ethical and GCP aspects of clinical trials of medicinal products for human use conducted outside of the EU/EEA and submitted in marketing authorization applications [27]; and European Commission guidelines on good clinical practice specific to advanced therapy medicinal products [22].

Issues related to both paediatrics and vulnerability and disability were found in almost all documents, mainly dealing with informed consent and ethics committee expertise/composition (Table 1). In contrast, no specific ethical issue on ATMPs is available.

**Table 1:** Regulatory/ethical/legal provisions applicable in the European Union, the research on inherited neurometabolic diseases

Document	Relevant topics	Specific provisions			
		Paediatric issues	Rare diseases	Inherited/genetic issues	Mental disability/vulnerability
Regulation (EU) 536/2014	Authorisation and conduct of trials, ethics committees, informed consent and assent process, vulnerability, minors, data protection and confidentiality, protocol	Art. 10, 32, 35 Recitals 19, 27	Recitals 9, 10		Art. 10, 28, 29, 31, 35 Recitals 15, 19, 27
Directive 2005/28/EC	Conduct of the trial, ethics committee	n.s.	n.s.	n.s.	n.s.
ICH Topic E 6 (R1) Guideline	Conduct of trials, informed consent, clinical trial protocol, ethics committee, vulnerability	Par. 4.8.12	n.s.	n.s.	Par. 1.61, 3.1.1, 4.8.12
EMA Reflection paper on clinical trials conducted outside EU/EEA	Conduct of multi-national trials, informed consent and assent process, ethics committee, confidentiality vulnerability, design of clinical trials	4.2, 4.3, 4.5, 5	5.1, 5.2		4.2, 4.5
EC guidelines on advanced therapy medicinal products	Conduct of trials with advanced therapies, clinical trial protocol	n.s.	n.s.	n.s.	n.s.
EU Charter of Fundamental rights	Children’s rights	Art. 24	n.s.	n.s.	n.s.
Declaration of Helsinki	Conduct of human research, vulnerability, risk/benefit, ethics committee, informed consent, privacy and confidentiality	19, 20, 28, 29	n.s.	n.s.	19, 20, 28, 29, 30
Oviedo Convention	Informed consent, subjects unable to give the consent, persons who have a mental disorder, subject’s rights			Art. 12	Art. 6, 7, 17
Additional Protocol to the Oviedo Convention	Risk/benefit, ethics committee, information and consent, privacy and confidentiality, vulnerability	Art. 15	n.s.	n.s.	Art. 15
Recommendation Rec(2006)4	Biological samples handling, information and consent, privacy and confidentiality	n.s.	n.s.	n.s.	n.s.
CIOMS-WHO guidelines 2002	Informed consent, clinical trial protocol, ethics committee, benefit/risk, vulnerability, privacy and confidentiality, secondary use of data	Guideline 14	n.s.	Guidelines 4, 5, 8, 18	Guidelines 4, 9, 13, 15
Directive 95/46/EC	Data protection and confidentiality, informed consent, subjects unable to give the consent, subject’s rights (art. 1-8; 10-34)	n.s.	n.s.	n.s.	n.s.
Directive 2001/83/EC	Data protection and retention, confidentiality (art. 21, 5.2.c)	n.s.	n.s.	n.s.	n.s.
Recommendation No. R (97) 5	Data protection and confidentiality, informed consent, subjects unable to give the consent, subject’s rights	n.s.	n.s.	4.7	12.2
Convention of 28 January 1981	Data protection and retention	n.s.	n.s.	n.s.	n.s.
UNESCO Declaration on Human Genetic Data, 2003	Informed consent, subjects unable to give the consent, biological samples, ethics committee, data protection and confidentiality	n.s.	n.s.	Whole document focused on genetic issues	n.s.
Recommendation No. R (92) 3	Informed consent, minors, persons suffering from mental disorders, data protection and handling, confidentiality	Principle 5	n.s.		Principle 5
ICH Topic E 11	Design and conduct of paediatric trials, assent, information, ethics committee	Whole document focused on paediatric issues	n.s.	n.s.	2.6.3
Ethical Recommendations, 2008	Design and conduct of paediatric trials, assent, information, ethics committee, risk/benefit, data protection, disclosure of genetic findings		6.1, 9.1	9.1, 18	
Paediatric Regulation (EC) 1901-1902/2006	Balance between the development of well-studied medicines and ethical concerns for enrolling young patients (Recital 7)		n.s.	n.s.	n.s.

*n.s.* not specified

Rare diseases are only cited in the new EU regulation [28] and EMA Reflection Paper [27], but no provision is established.

Among the other relevant international guidelines/documents and texts [9, 11, 12, 15, 16, 53], CIOMS-WHO guidelines [9] add specific details on the justification for enrolling ‘special populations’ like children and vulnerable patients, as well as on the informed consent and assent process. The recommendation is to seek a separate informed consent for the analysis of genetic material, if the genetic research is not a necessary part of the main clinical study. In addition, the use of results of genetic tests and familial genetic information and the precautions to prevent disclosure of the results of a subject’s genetic tests to immediate family relatives or to others are taken into account. The need for medical and psychological support for children and parents is also mentioned.

Further ethical provisions on data protection and confidentiality [10, 14, 29, 31] apply in the field of clinical research. However, no special requirements are available for issues taken into account in this work (i.e. paediatric and vulnerable patients, rare and genetic diseases). Council of Europe Committee of Ministers (CoE) recommendation on the protection of medical data [14] specifies rules to allow a clinical study even though personal data are not fully anonymised.

Specific documents deal with genetic tests and PGt/PGx research [13, 52]. They recommend (and do not make mandatory) that ad hoc consent is to be sought for genetic tests, thus meaning that a consent separate from the main study is appropriate in the case of clinical trials with genetic sub-studies. The consent should include information on subsequent processing, use and storage and indicate that the subject has the right to decide whether or not to be informed of the results [52].

Further guidance covering ethical aspects in the paediatric field can be found [21, 24, 32, 33]. In particular, ICH topic E11 [24] adds special restrictions when planning a paediatric clinical trial in ‘more vulnerable populations’ and handicapped or institutionalised paediatric populations. Rare diseases are mentioned in the EC Recommendations [21], as well as genetic issues, for which disclosure in clinical trials of genetic findings is recognised as a possible risk, thus requiring expert counselling in an adequate setting. The need for justifying alternative designs and/or analyses and agreeing these applications with competent authorities is mentioned. Table 1 summarises the provisions applicable in the iNMD field.

### The application of ethical provisions retrieved in the literature

We searched the literature in order to find the possible existing information on the application of ethical rules in iNMD clinical research and the current debate on the ethical issues (Table 2).

**Table 2:** Field of application, topics dealt and outcomes resulting from the bibliographic search

Source	Fields	Relevant topics	Recommendations/suggestions
Ross, 2010 [47]	Newborn genetic screening	- Use of stored samples for research - Parents’ informed consent	- To seek for the consent from parents for research on stored sample storage and research
Simopoulos, 2009 [50]	Newborn genetic screening	- Appropriateness of the investigation	- To protect individuals identified by genetic screening against the psychological and social hazards
Glantz et al., 2008 [38]	Biobanks	- Use of stored samples for research - Informed consent for secondary use - Owner of stored samples	- To establish clear rules on the use of samples in medical research and genetic privacy when information is used by companies - To provide participants the right to withdraw the consent
Botkin, 2005 [3]	Newborn genetic screening	- Use of stored samples for research - Appropriateness of the investigation	- To apply an approach to evaluate screening tests as rigorous as the approach for drugs (phases I to IV)
Sheela et al., 2005 [49]	Trials/experimental uses	- Parents’ informed consent - Vulnerable status - Approval from the ethics committee	- To provide full and appropriate information to parents - To offer adequate time for the parents to absorb information
Gelsinger, 2002 [35]	Trials/experimental uses	- Informed consent - Potential benefits, risks and discomforts	- To provide full and appropriate information - To carefully review paediatric protocols
Merz et al., 2002 [43]	Genetic research	- Use of stored samples for research - Parents’ informed consent - Rights of research participants and children	- To address issues on the control of research results and the sharing of benefits before the research is performed - To involve research participants in decision-making - To develop policies for protecting the interests of subjects
Clague, Thomas, 2002 [7]	Newborn genetic screening	- Secondary use of samples for research - Parents’ informed consent for secondary use - Right to privacy and anonymised data - Appropriateness of the investigation	- To undertake long-term storage, to allow re-evaluation of apparently erroneous results - To perform anonymously research
Pschera, 2000 [44]	Trials/experimental uses	- Appropriateness of the investigation	- To enhance the methodology for a safe and broad application of experimental approaches
Fox,. 2000 [34]	Trials/experimental uses	- Informed consent for gene therapy trials - Disclosure of conflicts of interest	- To provide full and appropriate information - To disclosure conflicts of interest
During, 1996 [18]	Trials/experimental uses	- Informed consent - Expertise in ethics committees	- To provide full and appropriate information - To foresee specific expertise in ethics committee on gene therapy

The literature search produced 50 records. The abstract/full text of two of them were not available. Eleven resulted compliant, i.e. dealing with ethical issues of research involving iNMD patients [3, 7, 18, 34, 35, 38, 43, 44, 47, 49, 50]. As shown in Table 3, these publications are set in several fields.

**Table 3:** Field of application, topics dealt and outcomes resulting from the European projects

Project	Fields	Relevant topics	Outcomes
BIONET [1]	Genetic research	- Ethical governance and regulation of research in China and EU	No relevant public information
COB [8]	Genetic research	- Cultural diversity and harmonisation of governance in Europe	No relevant public information—recommendations for policy makers not publicly available
Ethical, Legal and Social aspects of Brain Research [19]	Neurological disorders	- Ethical, legal and social aspects of brain research	- Need to define ethical and legal standards specifically applicable to brain research
InNerMeD-I-Network [40]	Rare inherited, paediatric, neurological diseases	- Ethical issues on clinical research, informed consent, vulnerable patients, regulatory, ethics committees	- Recommendations for researchers, ethics committees and patients to conduct studies involving iNMD patients with the highest ethical standards
LEUKOTREAT [41]	Rare inherited, paediatric, neurological diseases	- Informed consent - Ethical impacts of the proposed therapeutic challenges - Data sharing	- Identification of patient’ expectations towards research - A charter on data sharing within the project database - Recommendations on data processing, participant information - Template of information sheet for patients and families
RD-CONNECT [45]	Rare diseases	- Informed consent - Data protection - Data sharing - Registries - Biobanks	- Guidelines and standards for informed consent procedures, including essential elements to be dealt with and templates of prospective, retrospective and broad consent - Recommendations for researchers on the informed consent with specific information on data sharing - Appropriate time to think and ask questions for participants - To consult patients/participant representatives on the quality of information - Guidelines for effective and ethical data and sample sharing [42]
RESPECT [46]	Paediatrics	- Participation and empowerment of children in clinical trials, expectations and needs - Informed consent/assent	- Main barriers to participation of children in clinical trials identified - Recommendations for investigators, sponsors, patient organisations and policy makers
SCPE network [48]	Neurological paediatric diseases	- Informed consent	No relevant public information
TEDDY [51]	Paediatrics	- Informed consent/assent process - Minimal risks/burden - Emergency situations - Placebo use - Confidentiality and privacy, right to information - Contents of documents to be submitted to ethics committee - Compensation for damage	- To achieve agreed common definitions and procedures in the European Union with reference to genetic tests and studies - To adopt special measures, taking into account developmental needs of children at different ages and individual variations between children of the same age

Five out of 11 publications were laid down in the context of trials/experimental uses: three of them were focused on gene therapy [7, 18, 34], one showed results from the experimental treatment of an extemporaneous formulation administered to a paediatric patient [35] and one discussed about in utero stem cell transplantation [44].

Four publications were laid down in the context of newborn genetic screening programs [3, 7, 47, 50].

Finally, one publication was about biobanks [38], and another one was related to genetic research [49].

More in detail, different topics were examined as follows.

Five publications dealt with the use of stored samples for research purposes [3, 7, 38, 43, 47]. Specifically, Glantz and colleagues considered the issue on the ownership of the stored samples as well as rights of (paediatric) participants [38].

Eight publications dealt with informed consent issues [7, 18, 34, 35, 38, 43, 47, 49]. Three discussed about the consent from parents/legal guardians in the case of paediatric participants [7, 47, 49]. None mentioned the assent and the involvement of minors in the informed consent/assent procedure (in the case of neonatal screening [7, 47], we did not expect to find any information on this).

In addition, three publications explicitly considered the need for participant/legal guardian consent for the secondary use of data and samples [7, 38, 47].

The right to privacy and confidentiality was mentioned in three publications [7, 38, 50].

Three publications specifically discussed the interests and rights of children as research participants [7, 38, 43].

Finally, four publications argued about appropriateness of the experimental approach to be followed in order to protect individuals [3, 7, 44, 50].

Other topics were also discussed.

Sheela and colleagues considered the need for the ethics committee’s approval [49]. During highlighted the need for specific expertise in ethics committee assessing gene therapy trials [18]. Ross claimed the need for trained paediatricians [47]. Fox examined the need for disclosing conflicts of interest [34].

Seven publications (7/11) were about paediatric patients [3, 7, 38, 43, 47, 49, 50], most of them dealing with research in newborn screenings [3, 7, 47, 50].

Five publications faced ethical aspects related to genetic/inherited features of the diseases affecting research participants [3, 7, 47, 49, 50].

Two publications took into account the rarity of conditions [7, 50].

Only one publication discussed about the vulnerable status of an iNMD patient due to the frequently neurodegenerative nature of disease and about the potential impacts on unaffected family members [49].

No publication faced the balance between the development of well-studied and appropriate medicines in this field and the ethical concerns for enrolling young patients.

### Results from European projects

Additional information was derived from EU-funded projects. Fifty-one projects were found in CORDIS database. As none was found in CHAFEA, we screened 84 projects retrieved with ‘rare disease’ search string. Overall, nine projects resulted compliant, i.e. dealing with ethical issues of research involving rare or paediatric patients and genetics, focusing on different topics (Table 3).

Five projects considered paediatric issues [40, 41, 46, 48, 51], four of them discussed diseases with neurological impairment [19, 40, 41, 48], three were focused on rare diseases [40, 41, 45] and two on genetic research [1, 8].

The attitude, expectations and needs related to the participation and empowerment of children in clinical trials were explored in COB [8], InNerMeD [40], LEUKOTREAT [41], RESPECT [46] and TEDDY [51] projects. In detail, the analysis performed within RESPECT indicated four main barriers to participation of children in clinical trials: emotional barriers, ethics and transparency of information, practical barriers and the relationship with physicians. The project also identified several needs for the participants: to be treated as a partner in the clinical trial process, to access information about the clinical trial, to increase the patients’ ability to play an active role in the decision-making process, to develop an educational programme for patient organisations in order to increase their knowledge of clinical trials and to act as a partner in the clinical trial process, to increase ethics committees’ competence and involvement in paediatric research and their networking, to fulfil the need for greater transparency and access to trial results in order to prevent unnecessary paediatric clinical trials throughout Europe and to provide patient organisations a more active role before, during and after the implementation of a clinical trial.

Informed consent procedures were dealt with five projects [40, 45, 46, 48, 51], three also including assent procedures [40, 46, 51].

Regulatory/ethical/legal aspects were part of seven projects [1, 8, 19, 40, 41, 45, 51]. In particular, LEUKOTREAT [41], which specifically deals with a group of iNMDs, sets out the basis of its work in the Declaration of Helsinki [53] and CIOMS-WHO Ethical Guidelines [9]; TEDDY [51] and InNerMeD [40] on the EC Recommendations [21], ICH Topic E11 [24] and the other relevant guidelines. In particular, TEDDY “Ethical recommendation on pharmacogenetic/genomic research” endorsed that a common position is shared among stakeholders, considering the type of information given to patients and parents/legal representatives, especially when neonates, who cannot participate in the informed consent/assent process, are involved; procedures and expertise to be included in the ethics committee when genetic tests and studies are concerned; access to biological materials and data stored in biobanks in lack of consent or assent; time of archiving genetic material in biobanks; access to genetic results from parents/careers according to CIOMS-WHO Ethical Guidelines [9]. These recommendations were implemented in the InNerMeD-I-Network [40] project.

Data protection/confidentiality issues including data sharing among different centres or countries were found in two projects [41, 45]: the increasing relevance of data sharing among international research consortia, and the need for coordination and harmonisation between different research centres, the right to benefit from research, the preservation of identity and privacy for rare disease patients are dealt within RD-CONNECT project [45]. Importantly, the use of prospective, retrospective and broad consent, re-consent, waiver of consent, opt out method (giving consent by not declining to give consent) is discussed and templates are provided.

## Discussion

The European legal framework offers a number of ethical provisions ruling the clinical research in general as well as specifically applicable to the iNMDs field, i.e. rare inherited diseases with neurological symptoms often involving young and very young patients.

From a general point of view, in Europe, all clinical trials follow the same assessment and authorisation. From 2016, with the entry into force of the new Regulation (EU) 536/2014 [28], a unique central procedure will be in place through a unique portal by submitting a homogeneous package for all Member States. This includes specific conditions to begin clinical research involving vulnerable persons, i.e. minors (art. 32) and incapacitated subjects (art. 31). For paediatric trials, the assessment must take into account PDCO opinions on Paediatric Investigation Plans (PIPs) and must be carried out by ethics committees with paediatric expertise or seeking advice from experts in the field taking into account the risks and benefits of research and other aspects such as the study design, the use of placebo and the monitoring of safety during and after the trial. This represents an important achievement from the ethical point of view and in consideration of the protection of children’s rights [37]. However, uncertainties still remain on how national ethics committees and the PDCO will interact and what would happen if the PDCO and the ethics committee opinions differ [36]. Therefore, given that ethics committees and PDCO evaluate many common elements while having distinct roles and responsibilities, the applications should be harmonised and standardised.

On the basis of our results, we can derive the following considerations regarding the above-mentioned features of iNMDs.

Most of the regulatory documents do not deal with ethical aspects related to *rare diseases*. Unfortunately, the new clinical trial Regulation (EU) No 536/2014 [28] only mentions the importance of clinical trials and timely availability of drugs for patients with rare and ultra-rare diseases in two recitals, but it does not establish applicable provisions. In fact, it does not make mandatory either a faster assessment of these trials or the involvement of specific expertise in the assessment phase. In line with the regulatory findings, ethical aspects related to the rarity of iNMDs are poorly dealt with in the literature, even though several EU projects are focused on rare diseases.

Specific ethical/regulatory documents are available for *paediatric research*. The recommendations released by the European Commission in 2008 [21] include the highest standard to be met. They deal with the benefit and risk balance, the process of information and consent/assent according to an age-staggered approach, the ethical review of paediatric protocols, the individual data protection and insurance issues. They mention that children with chronic illness may have increased capacity to make independent judgements based on previous life experience. Currently, this document is under revision and the European Commission launched a public consultation (from 1 June 2016 to 31 August 2016) to seek the views of stakeholders in preparation for the implementation for the new Clinical Trials Regulation (EU) No 536/2014.

However, as underlined in this work and in line with previous considerations [37], they have a poor legislative power and are implemented only partially in the regulation recently released [28].

With regard to the *informed consent and assent*, it has been recognised that before the decision to participate or abstain from participation, children are entitled to receive oral and written information according to age and level of maturity [21, 24, 28]. However, the existing provisions on informed assent are not fully applicable to iNMDs and are not enough, as the *neurological impairment* often leads to a different mental maturity of patients, both adult and paediatric, and makes them ‘vulnerable persons’ unable to provide their consent. As demonstrated by the analysis performed, at a regulatory level, this concept has been implemented in different documents [9, 11, 13, 14, 21, 24, 25, 27, 28, 53] and the need for obtaining the consent from a legal representative is made mandatory from the EU Regulation [28].

Other ethical issues arising great interest in the scientific community, as demonstrated in the regulatory framework, the literature and EU projects, include *genetic aspects*, data protection, confidentiality and the handling of biological samples, including the ‘secondary’ use of stored samples for research purposes after testing is completed. The existing provisions applicable in Europe appear highly relevant as they suggest procedures to safeguard the rights of research participants [9, 13, 52]. However, also these documents have a poor legislative power and their application requires a great effort from researchers, especially in the case of multi-centre and multi-national studies in which national laws also have to be complied with. Importantly, it should be noted that these issues have not ad hoc rules in paediatrics.

The analysis performed in the literature reveals that a great part of publications dealt with informed consent issues, but none mentioned the assent and the involvement of minors in the informed consent/assent procedure.

Finally, even though the literature provides a number of recommendations/suggestions addressed to different stakeholders (researchers/clinicians, policymakers/health institutions, ethics committees, patients and their families), including the implementation of clear ethical rules [38, 50], no publication considers the application of regulatory/ethical/legal provisions in the iNMD field, either in Europe or in any country in the world.

Additional relevant information was found in *EU*-*funded projects*. Rare inherited paediatric neurological disorders are the focus of InNerMeD-I-Network [40] and LEUKOTREAT [41] projects.

Because of the paucity of current information about most of these disorders, the ‘Inherited NeuroMetabolic Disease Information Network’ project was launched with the aim of creating the first European network of information related to diagnosis and treatment of iNMDs. One of the goals of the project was to straighten research capacities in the iNMD field and to overcome the existing barriers for developing drugs for small populations. In this context, methodological and ethical recommendations have been publicly released for researchers, including companies and research centres, ethics committees, patients and families to perform well-conducted research involving iNMD patients.

Also, TEDDY [51] and RESPECT [46] FP projects released recommendations on ethical issues in paediatrics with the aims to fill the gap of the regulatory framework and to improve information to stakeholders and patients/families on issues related to paediatric research, such as the informed consent and assent process. Furthermore, the GRiP project (Global Research in Paediatrics - 261060 - FP7-HEALTH-F5-2010) provided documents on the ethical review of multi-jurisdictional trials and models for information sharing on human research among ethics committees and Institutional Review Boards in the paediatric field.

Another important issue raised is the relevance of sharing patient data from different countries from the ethical point of view [41, 45]. This is increasingly widespread in the case of rare disease and particularly challenging when these data are from children and include genetic data.

Importantly, no specific ethical issue on the use of ATMPs for iNMDs and paediatric rare diseases in general is available in the regulatory framework. This claims the need for further specific guidelines/recommendations in this field that could be particularly relevant to iNMDs.

The lack of substantial guidance from the legal and methodological point of view emerges in the regulatory framework, as demonstrated in the literature and underlined in the framework of research projects.

Overall, these EU projects underline the interest from the European Commission in the field. In the framework of the new research programme Horizon 2020, a project on the study of the changes deriving from the application of the new Regulation on clinical trial [28] and the proposed Regulation on privacy and data protection [23] might be expected.

Conclusively, this work highlights the need for a common ethical approach to the clinical research involving iNMD patients and, more in general, rare neurological diseases also affecting children. This approach should represent the basis for developing official ethical recommendations for researchers, companies, ethics Committees, patients and families to be shared with regulatory agencies and public health bodies.

## Abbreviations

European Medicines Agency: (EMA)
Inherited NeuroMetabolic Disease: (iNMD)
